# The Dual Role of Macropinocytosis in Cancers: Promoting Growth and Inducing Methuosis to Participate in Anticancer Therapies as Targets

**DOI:** 10.3389/fonc.2020.570108

**Published:** 2021-01-19

**Authors:** Shaojuan Song, Yanan Zhang, Tingting Ding, Ning Ji, Hang Zhao

**Affiliations:** State Key Laboratory of Oral Diseases, National Clinical Research Center for Oral Diseases, Chinese Academy of Medical Sciences Research Unit of Oral Carcinogenesis and Management, West China Hospital of Stomatology, Sichuan University, Chengdu, China

**Keywords:** macropinocytosis, promoting cancer growth, methuosis, anticancer therapies, extracellular proteins, anticancer drugs delivery

## Abstract

Macropinocytosis is an important mechanism of internalizing extracellular materials and dissolved molecules in eukaryotic cells. Macropinocytosis has a dual effect on cancer cells. On the one hand, cells expressing RAS genes (such as K-RAS, H-RAS) under the stress of nutrient deficiency can spontaneously produce constitutive macropinocytosis to promote the growth of cancer cells by internalization of extracellular nutrients (like proteins), receptors, and extracellular vesicles(EVs). On the other hand, abnormal expression of RAS genes and drug treatment (such as MOMIPP) can induce a novel cell death associated with hyperactivated macropinocytosis: methuosis. Based on the dual effect, there is immense potential for designing anticancer therapies that target macropinocytosis in cancer cells. In view of the fact that there has been little review of the dual effect of macropinocytosis in cancer cells, herein, we systematically review the general process of macropinocytosis, its specific manifestation in cancer cells, and its application in cancer treatment, including anticancer drug delivery and destruction of macropinocytosis. This review aims to serve as a reference for studying macropinocytosis in cancers and designing macropinocytosis-targeting anticancer drugs in the future.

## Introduction

Macropinocytosis is a non-selective liquid-phase endocytic pathway for the uptake of extracellular substances. Recently, relationship between macropinocytosis and cancers has attracted increasing attention. The process of macropinocytosis was first demonstrated by Warren H. Lewis using time-lapse video cinematography in 1931, and he coined the term “pinocytosis”, or “cell drinking” (1). Next, in 1986 and 1992, Bar-Sagi and Ridley et al. described the induction of membrane ruffling and fluid-phase pinocytosis by Ras and Rac protein (2, 3). And a review on macropinocytosis was published in 1995 (4). Later, Chi et al. found that abnormal expression of RAS genes in gastric cancer and glioma cells would cause cellular degeneration and vacuolization (5). This vacuolization belongs to macropinocytosis and eventually led to a new form of cell death different from apoptosis. Since then, there have been endless researches on the molecular mechanism of macropinocytosis in tumor cells and dendritic cells (DCs). The regulatory factors involved in macropinocytosis include ADP ribosylation factor-6 (Arf6) (6), actinin-4 (7), p21-activated kinase 1 (PAK1) (8), Cdc42 (9), Rac (10), Rab GTPases (11, 12), RhoA (13), and phosphoinositides(PIs) (14). Particularly, Kaul et al. discovered that cell vacuolation and cell death in glioblastoma (GBM) may experience a unique novel cellular pathway (15). This cellular pathway involves unique molecular loci that could be exploited to cause apoptosis-independent cell death of cancers, which was termed ‘methuosis’ from Greek “methuo” (to drink to intoxication) by Overmeyer et al. (16).

**Figure 1:**
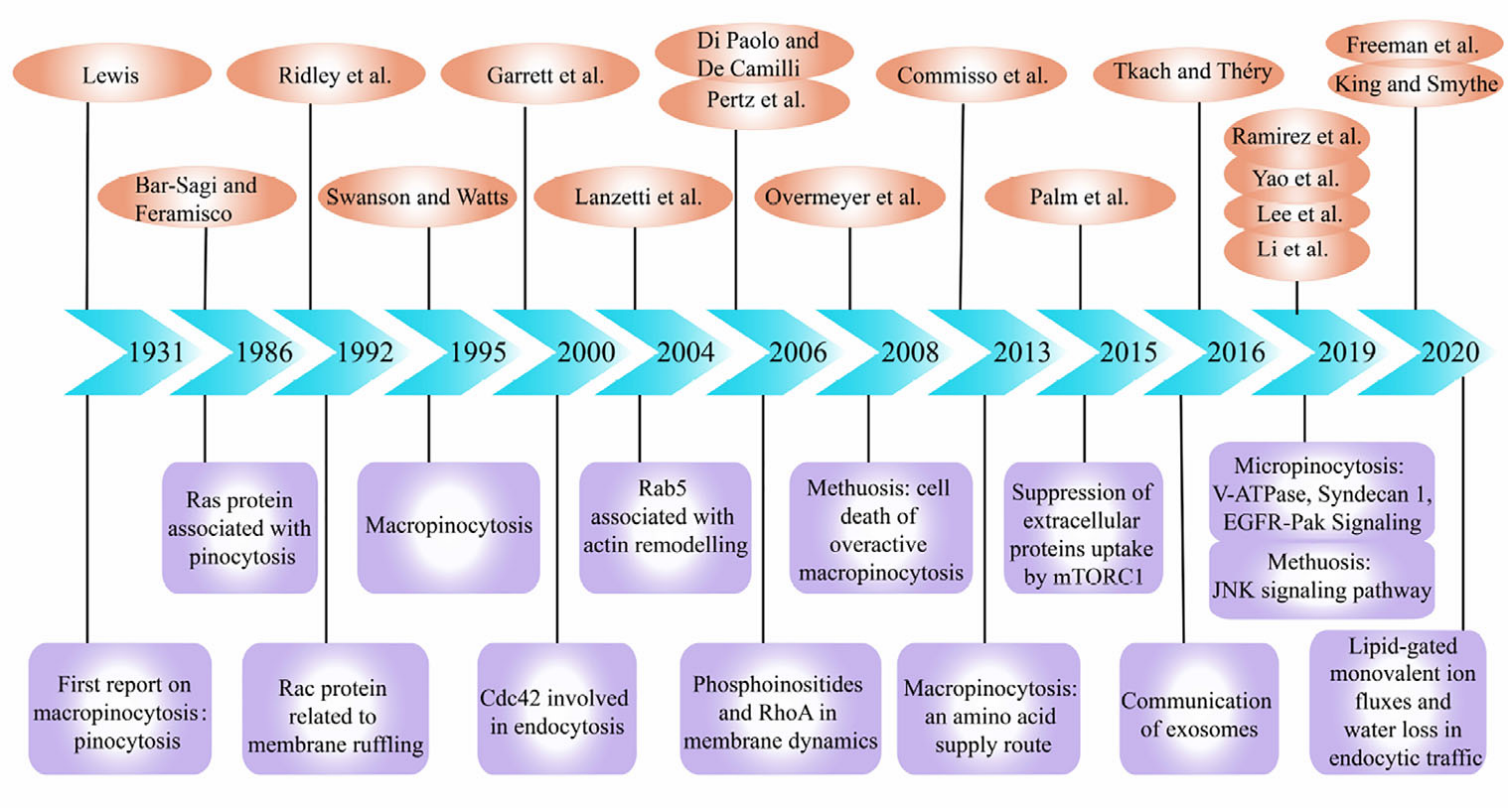
Important events during research development of macropinocytosis in cancers. The references and themes are as follows: Pinocytosis. (1) Induction of membrane ruffling and fluid-phase pinocytosis in quiescent fibroblasts by Ras proteins. (2) The small GTP-binding protein rac regulates growth factor-induced membrane ruffling.(3) Macropinocytosis.(4) Developmental control of endocytosis in dendritic cells by Cdc42 (9). Rab5 is a signaling GTPase involved in actin remodeling by receptor tyrosine kinases (12). Phosphoinositides in cell regulation and membrane dynamics (14). Spatiotemporal dynamics of RhoA activity in migrating cells (13). Active RAS triggers death in glioblastoma cells through hyperstimulation of macropinocytosis (16). Macropinocytosis of protein is an amino acid supply route in RAS-transformed cells (17). The Utilization of Extracellular Proteins as Nutrients Is Suppressed by mTORC1 (19). Communication by Extracellular Vesicles: Where We Are and Where We Need to Go (20). EGFR-Pak Signaling Selectively Regulates Glutamine Deprivation-Induced Macropinocytosis (25). Plasma membrane V-ATPase controls oncogenic RAS-induced macropinocytosis (22). Syndecan 1 is a critical mediator of macropinocytosis in pancreatic cancer (23). The JNK signaling pathway plays a key role in methuosis (non-apoptotic cell death) induced by MOMIPP in glioblastoma (26). Lipid-gated monovalent ion fluxes regulate endocytic traffic and support immune surveillance (27). Water loss regulates cell and vesicle volume (28).

Next, the researchers discovered that in addition to participating in methuosis, macropinocytosis plays a role in promoting cell growth in certain cancer cells. In 2013, Commisso et al. reported that K-RAS-transformed cells supply themselves with amino acids by proteins internalization through macropinocytosis (17). Similarly, Qian et al. found that cancer cells can increase their own ATP levels by internalizing extracellular ATP (eATP) through macropinocytosis in 2014 (18). In 2015, Palm et al. reported that when cancer cells rely on the internalization of extracellular proteins to obtain amino acids, stimulation to the mechanistic target of rapamycin compound 1 (mTORC1) could inhibit cell proliferation, as opposed to promoting cell proliferation when amino acids are abundant (19). Also, the transport of exosomes was reported to be related to macropinocytosis in 2016 (20). In particular, Kim et al. discovered that macropinocytosis can promote the survival of malnourished PTEN-deficient prostate cancer cells (21). In recent years, studies have reported that plasma membrane vacuolar ATPase (V-ATPase) (22), Syndecan 1 (SDC1) (23), mTORC1/mTORC2 (24), and epidermal growth factor receptor (EGFR) pathways (25) are closely associated with the molecular mechanism of macropinocytosis in tumor cells. In addition, the C-Jun N-terminal kinase (JNK) signaling pathway played a key role in methuosis (26). The latest research shows that lipid-gated monovalent ion fluxes and water loss also play a role in the endocytosis pathway including macropinocytosis (27, 28) (**Figure 1**).

Macropinocytosis has a dual role on cancers. It may enhance the invasion of cancers under certain conditions such as starvation through increased extracellular nutrient supplies, circulating of plasma membranes and receptors such as the death receptors (DRs), ErbB3, platelet-derived growth factor β-receptor (PDGFRβ), EGFR, and neonatal Fc receptor (FcRn), and EVs internalization. Nutritional supply is not only reflected in the fact that macropinocytosis can provide extracellular proteins to cancer cells during nutrient deficiency states and convert them into amino acids, such as glutamine, to maintain their viability, but is also reflected in the internalization of other nutrients, including carbohydrates, fats, nucleotides, and eATP. Hyperactivated macropinocytosis caused by certain abnormally activated genes such as K-RAS and certain specific small molecule drugs such as MIPP may induce a novel form of cell death distinct from apoptosis: methuosis. Based on the above characteristics, several researchers are committed to making macropinocytosis a new target for cancer treatment. Macropinocytosis has excellent potential for delivering anticancer drugs. At the same time, the design of anticancer drugs that can induce methuosis or abrogate the process of macropinocytosis have also been reported. All these are important research findings in cancer treatment. Herein, this review covers the following four sections: the general process, beneficial effects (promoting cancer growth), harmful effects (methuosis), and anticancer therapies targeting macropinocytosis in cancer cells.

## The General Process of Macropinocytosis

**Figure 2:**
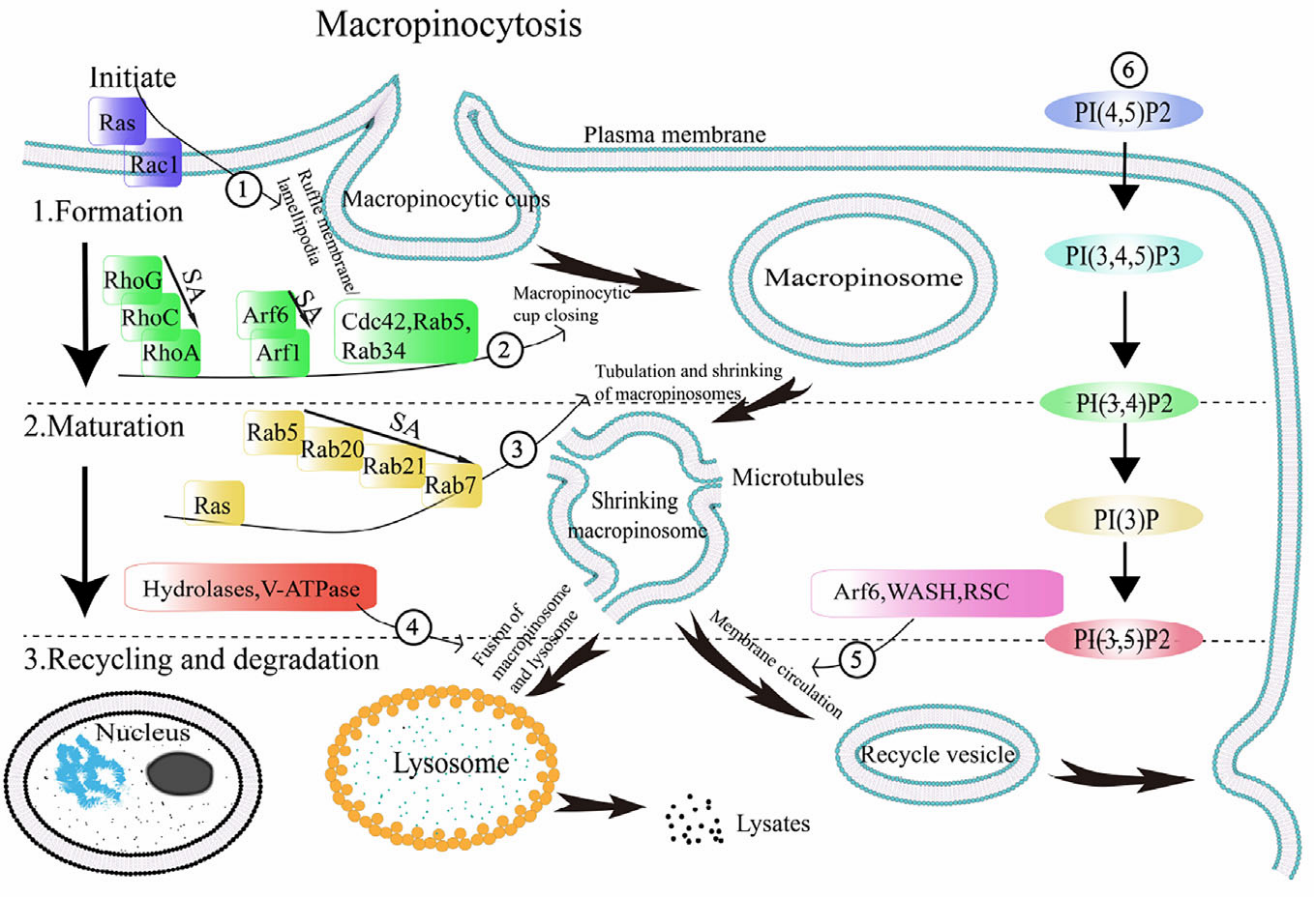
General process of macropinocytosis. The regulatory factors involved in the process of macropinocytosis are shown in ①-⑥. 1. Macropinosome formation: under the activation of the starting protein Ras, ruffle membrane/lamellipodia is formed, and further developed into macropinocytic cups. Through the sequential activation of a series of related small GTPases and phosphoinositides (PIs), the macropinocytic cups are closed to form macropinosomes. 2. Macropinosome maturation: the sequential activation of a series of related small GTPases and PIs is involved in regulating the occurrence of macropinosome tubulating and shrinking, so that the macropinosome enters a mature state. 3. Degradation and recycle: macropinosomes partially cycle back to the plasma membrane surface and fuse with the membrane, while others fuse with lysosomes and become cleaved. SA: sequential activation. Arf1/6:ADP ribosylation factor-1/6, V-ATPase: vacuolar ATPase, WASH: WASP and SCAR homologues, RSC: retromer sorting complex, PI(4,5)P2: phosphatidylinositol 4,5-bisphosphate, PI(3,4,5)P3: phosphatidylinositol 3,4,5-triphosphate, PI(3,4)P2: phosphatidylinositol 3,4-bisphosphate, PI(3)P: phosphatidylinositol-3-phosphate, PI(3,5)P2: phosphatidylinositol 3,5-bisphosphate.

Endocytosis includes two mechanisms: clathrin-dependent and clathrin-independent endocytosis. The vesicles formed during clathrin-dependent endocytic mechanisms have a small diameter (~ 100 nm), which has been reported in the literature as micropinocytosis, and the vesicles derived from the envelope are called endosomes (4). The vesicles formed during clathrin-independent mechanisms have a larger diameter (> 0.2–5μm), which is approximately 1,000 times larger than the endosomes (29). This process is called macropinocytosis (30), and the vesicles formed in this process are called macropinosomes (31). Macropinocytosis occurs during pathogen-host interaction as well as in cancers. Although there are some differences in the regulation and effect of macropinocytosis in various cell types and diseases, the general process is essentially the same. In most cells, macropinocytosis is mediated and regulated by a series of receptors and ligands that have a specific order and location in time and space (14). Here, we introduce the three stages of macropinocytosis (the formation, maturation, and recycling and degradation of macropinosomes) and the major regulators (small GTPases and PIs) (**Table 1**) involved in the general process of macropinocytosis (32–34) (**Figure 2**).

**Table 1:** The classification and functions of the regulatory factors involved in macropinocytosis process.

Regulatory factors		The relevant function
**Rho GTPases**	Rho : RhoA,RhoC,RhoG	Being activated and playing a role in the formation of macropinosomes.
	Cdc42	Combing with GTP to activate PAK1 to regulate actin in the cytoskeleton.
	Rac: Rac1	The sequential activation of Rac1 followed by inactivation promoting the formation of macropinosomes.
**Ras GTPases**	Ras	Ras activation stimulates the formation of macropinosomes, and the peak of Ras activity occurs after the formation of macropinosomes.
**Arf GTPases**	Arf1	Regulating actin cytoskeleton remodeling.
	Arf6	The presence of Arf6 has a guiding effect on the location of activated Rac1 on the plasma membrane.
**Rab GTPases**	Rab5	Regulating the actin skeleton remodeling and stabilizing macropinosomes and is one of the markers of early macropinosomes.
	Rab7	One of the markers of late macropinospmes.
	Rab20, Rab21	They function at the maturity stage of macropinosomes.
	Rab34	Promoting the formation of membrane ruffles.
**PI**	PI(4,5)P2	Binding to a variety of actin, activating actin, regulating actin polymerization, and promoting the shrinkage of the plasma membrane.
	PI(3,4,5)P3, PI(3,4)P2	Binding with Akt, Btk, PDK1, ARNO, and other proteins to activate downstream signaling pathways and regulating the depolymerization and reconstruction of actin.
	PI(3)P	Localizing on the macroponosomes membrane and the tubule membrane extending from macroponosomes and controlling early endosomes.
	PI(3,5)P2	Control late endolysosomes.

### Formation

The formation of ruffle membrane/lamellipodia marks the beginning of macropinocytosis (4). Some ruffle membrane/lamellipodia form a cup shape with round frills —macropinocytic cups (35). Subsequently, the macropinocytic cups close, indicating the formation of macropinosomes (36). In the above process, cytoskeleton remodeling is one important step. Macropinocytosis is an example of cellular compartment based on the changes of cytoskeleton interface that related to the neurofibromatosis type 2 (NF2) tumor suppressor merlin and cytoskeleton-linking protein ezrin (37). When the cytoskeleton remodeling is damaged or disturbed, it will affect the formation of macropinosomes. Disruption of actin or microtubules in DCs with specific inhibitors significantly prevented macropinocytosis (38). Meanwhile, SLIT2, a Slit protein, was found to inhibit macropinocytosis by opposing cortical cytoskeletal remodeling (39). Ruffle membrane/lamellipodia form macropinocytic cups, and then form macropinosomes, involving a series of small GTPases and PIs (**Figure 2**.)

The small GTPases involved in the formation of macropinosomes are mainly Ras, Rho family, Rab family, and Arf GTPases family. Remodeling of the actin backbone is the basis of macropinocytosis. The direct link between Ras and macropinocytosis was first identified based on the expression of activated oncogenic Ras proteins in fibroblasts, and ruffling was found to be activated accordingly (2). Ras proteins participate in the formation of macropinocytic cups by activating phosphatidylinositide 3-kinase (PI3K). In addition to activating PI3K, Ras can directly regulate cytoskeletal proteins, formin-C (ForC) is an example (40).

The Rho GTPase family participates in the regulation of macropinocytosis through active and inactive GTP-binding (41). The proteins playing vital roles in the adjustment of actin skeleton remodeling during macropinocytosis mainly include three groups, Rho, Cdc42, and Rac. The precise role of Rho protein in macropinocytosis has not been fully defined, although some researchers have found that Rho worked in the regulation of macropinocytosis. Zawistowski et al. reported that a subtype of Rho, RhoC, was temporarily activated before the macropinocytic cups closed to form macropinosomes (42). Ellerbroek, Samson, and others found that RhoG, also a subtype of Rho, played an significant part in the formation of macropinocytic cups in fibroblasts and A431 cells (43, 44). Pertz et al. also observed that after the macropinocytic cups closed to form macropinosomes, the activity of another Rho subtype, RhoA, increased abruptly (13). Although what role does Cdc42 protein plays during macropinocytosis remain unclear, some studies have reported its involvement in macropinocytosis activities in some cell types (45). Dharmawardhane et al. found that Cdc42, along with GTP, could activate PAK1 (46), which has a significant role in plasma membrane shrinkage and formation of macropinosomes during macropinocytosis (8). Rac1, one important subtype of Rac, has an irreplaceable role in the formation of macropinosomes. Previous studies have focused on the relationship between Rac1 activation and macropinosomes. Fujii et al. used optogenetics to regulate Rac1 activity to analyze the role of Rac1 activation and inactivation during macropinocytosis (47). They found that while Rac1 activation promoted the formation of macropinocytic cups in the plasma membrane, Rac1 inactivation was required when the macropinocytic cups closed to form macropinosomes. This suggests that the sequential activation of Rac1, followed by inactivation, is crucial for the formation of macropinosomes. Likewise, Rac is demanded for macropinocytosis which establishes the link between macropinocytosis and the cytoskeleton by activating actin groups (47).

Rab GTPases play a key role in regulating cell membrane transport during macropinocytosis (48). The sequential activation in different isoforms of Rab proteins is of great significance in the process of membrane transfer (49). It has been reported that once Rab5 is activated and aggregates on the cytoplasmic membrane, phosphatidylinositol 3,4,5-triphosphate (PI (3,4,5) P3) is produced, in which their synergy induces macropinosomes formation (50). At the same time, with the support of the corresponding effector Rabankyrin-5 (51), Rab5 regulates actin remodeling and participates in the formation of macropinosomes (12). Feliciano et al. observed that during macropinosome formation, Rab5a was activated and recruited into the macropinosomes (52). It reached a peak of activity and then began to decline before the macropinosomes were cleaved. It is speculated that Rab5 not only affects the regulation of actin skeleton remodeling, but also stabilizes macropinosomes. Rab34 also participates in the formation of macropinosomes. With the synergistic effect of Rac1 as well as the actin nucleating molecule WAVE2 (53), Rab34 promotes the formation of membrane ruffles.

The main role the Arf GTPases family is to regulate the metabolism of phospholipids and remodel the actin skeleton to regulate cell membrane transport activities. In the Arf family, the process of macropinocytosis are closely associated with the Arf6 and Arf1 proteins (54, 55). Arf6 was reported to have a guiding effect on the location of activated Rac1 on the plasma membrane (56). After activation, Arf6 caused the accumulation of ARNO (Arf guanine nucleotide exchange factor GEF), excited Arf1, and regulated actin cytoskeleton remodeling. Williamson et al. found that Arf6-GFP was present at the plasma membrane and on the peripheral macropinosomes that contained incoming cargo proteins (57), whereas the expression of a GTP-binding-defective mutant of Arf6, Arf6T27N-GFP, inhibited macropinocytosis, as was observed in H-RAS-expressing HeLa cells (50).

The PIs involved in the formation of macropinosomes are phosphatidylinositol 4,5-bisphosphate (PI(4,5)P2), phosphatidylinositol 3,4,5-triphosphate (PI(3,4,5)P3) and phosphatidylinositol 3,4-bisphosphate (PI(3,4)P2) in the order of their participation. When the plasma membrane ruffles occurs, PI(4,5)P2 begins to exert its function. Fluid uptake is dependent on phosphatidylinositol 4-phosphate 5-kinase (PI4P5K) required in the composition of PI(4,5)P2 (58). Araki, Welliver and Swanson detected high levels of PI(4,5)P2 in the ruffle membrane/lamellipodia and macropinocytic cups using live-cell imaging (59). PI(4,5)P2 binds to a variety of actins, activates actin, regulates actin polymerization, and promotes the shrinkage of the plasma membrane. In the macropinocytic cups in macrophages, the peak of PI(4,5)P2 level appears before PI(3,4,5)P3 (59). Araki, Yoshida, and Welliver and Swanson found that the level of PI(3,4,5)P3 increased significantly following macropinocytic cups formation during macropinocytosis by fluorescent probe technology (59, 60). The mechanism of action of PI(3,4,5)P3 is mediated through binding with Akt, Btk, PDK1, ARNO, and other proteins to activate downstream signaling pathways and regulate the depolymerization and reconstruction of actin (61). After closure of the cups, PI(3,4,5)P3 rapidly disappears from the vesicles and is substituted by PI(3,4)P2 (62).

### Maturation

Macropinosomes begin to mature following formation. (**Figure 2**). Tubulation and shrinking of macropinosomes is the step one of maturation (63, 64). Meanwhile, vesicles become more concentrated, suggesting that the contraction of the macropinosomes is not due to division but to missing of liquid and membrane. This may be caused by an increase in osmotic pressure, which causes them losing more superficial area than bulk from the vesicles during tubulation and fission (65). Shrinkage and concentration, therefore, are likely to be pervasive stages of macropinosome maturation. It appears to be reasonable that the formation of a smaller, more centralized space facilitates better assimilation.

The small GTPases involved in the mature stage of macropinosomes mainly include Ras and Rab. Ras participates in the transition process from macropinosome formation to maturity. Welliver and Swanson found that activation of Ras occurred after the formation of cup-shaped ruffles in the plasma membrane during macropinocytosis (59). They measured the degree of Ras activation and found that the peak value of Ras activity occurred after macropinosome formation. The canonical Ras signaling pathways are as follows: the Raf—extracellular signal-regulated kinase (ERK)—ERK cascade, PI3K—Akt pathway, and RalGDS—RalA/RalB pathway (15).

The sequential activation and inactivation of Rab proteins is one of the important conditions for the formation and maturation of macropinosomes (66, 67). When Rab proteins are recruited to the macropinosomes, Rab5 is the first protein to aggregate, followed by Rab21, and finally Rab7 (68). After entering the cells, vesicles generated by macropinocytosis immediately aggregate phosphatidylinositol-3-phosphate (PI(3)P) and activate Rab5, thus forming an “early” chamber. Then Rab5 is substituted with activated Rab7, and the macropinosomes enter the “late” phase including maturity and incorporation with lysosome (36). Rab5 acts as a universal label for emerging vacuoles. (**Figure 2**). Rab7 is a marker of late endosomes (64), and the presence of Rab7 have been detected in mature macropinosomes. This shows that mature macropinosomes share some similarities with late endosomes. After the dissociation of Rab5 and Rab21, Rab20 can still be found in macropinosomes, Rab20 and Rab21 appear on mature macropinocytosis (69). Almost all the Rab protein subtypes mentioned above are codomain in macropinosomes.

The PIs involved in the mature stage of macropinosomes are phosphatidylinositol-3-phosphate (PI(3)P) and phosphatidylinositol 3,5-bisphosphate (PI(3,5)P2) in order. Once the macropinosomes are formed, they enter the mature stage, during which PI(3)P makes significant contribution. During this stage, a mass of PI(3)P is discovered in its membrane. Yoshida et al. detected the transient presence of PI(3)P on the macropinosome membranes in macrophages through live-cell imaging (70). Araki et al. also found PI(3)P persisting on the macropinosome membranes of A431 cells (71). PI(3)P activation mechanism involves its combination to the PX domain of the sorting nexin 5 (SNX5), and localization on the macropinosome and tubule membranes extending from macropinosomes (72). At right time, the tubules detach from the macropinosomes and the macropinosomes reach maturity. In mammalian cells, shrinkage of macropinosome is partly regulated by the activity of phosphoinositide kinase, FYVE-type zinc finger containing (PIKfyve) protein, which phosphorylates PI(3)P to form PI(3,5)P2 (63). PI(3)P and PI(3,5)P2, which are essential during macropinosome maturation, regulate early and late macropinosome formation, respectively.

### Recycling and Degradation

After maturation, the newly formed macropinosomes undergo two processes mediated by different receptors: recycling and degradation (**Figure 2**). Freeman et al. demonstrated that sodium ions outflow mediated by two-pore channel (TPC) can lead to rapid volume loss of newly formed macropinosomes (27). TPC reduces the hydrostatic pressure inside the macropinosome, promotes the extension of microtubules from the surface of the macropinosome membrane, and further promotes the cycle process of proteins and membrane lipids returning to the cell surface (28). Surface proteins are quickly recovered after being internalized by macropinosomes to avoid degradation (22). This is due to the synergistic activities of WASP and SCAR homologues (WASH) and the retromer sorting complex (RSC) (73). These complexes are the first-line molecule substances which absorbed by phagosome/macropinosome and are strongly activated within the first 2–3 min after internalization.

Their function is involved not only in sorting proteins into circulating vesicles, but also in other aspects of the macropinocytosis process. In macrophages, macropinosomes contract, undergo early and late macropinosome stages, and eventually appear to fuse with and be completely assimilated by the lysosomal system (74). While the digestible components are transported out for cell assimilation, other molecule substances, like fluorescent dextra for macropinocytosis studying, are eventually liberated from the cell. Indigestible materials are continuously released by constitutive exocytosis (75). The second period of WASH activation gets rid of V-ATPase and proteolytic enzyme, thus promoting the transition of vesicles to a litmusless post-lysosome phase (76, 77). In macrophages, the release of the contents of macropinosomes undergoes complex membrane dynamics that may involving at least two pathways, which is coordinated with the complex redistribution of macrophages across multiple chambers (78). (**Figure 2**). In addition, De Lartigue et al. proposed that PIKfyve inhibition might block the fusion of late endosome/lysosome with EGFR-containing macropinosomes (79). TRPML1 is a Ca^(2+)^ channel in lysosome controled by PI(3,5)P2. Macropinosomes separated from TRPML1 silencing cells can be modified by lysosomes but do not fuse with lysosomes (80). This shows that PIKfyve and PI(3,5)P2 are also involved in the degradation of macropinosomes.

In short, the general process of macropinocytosis mainly involves cytoplasmic membrane shrinkage, formation of macropinocytic cups, generation of macropinosomes, maturation of macropinosomes, fusion of macropinosomes with lysosomes, and recycling and degradation.

## The Beneficial Effects of Macropinocytosis in Cancers—Promoting Cancer Growth

In recent years, researchers have increasing focused on the role of macropinocytosis in tumor cells, especially benefits. This part mainly summarizes the substances that can be internalized by macropinocytosis to promote the survival of cancer cells, including extracellular proteins (for amino acids), other nutrients, desired cell surface receptors, and beneficial EVs.

### Extracellular Proteins Internalization Through Macropinocytosis

**Figure 3:**
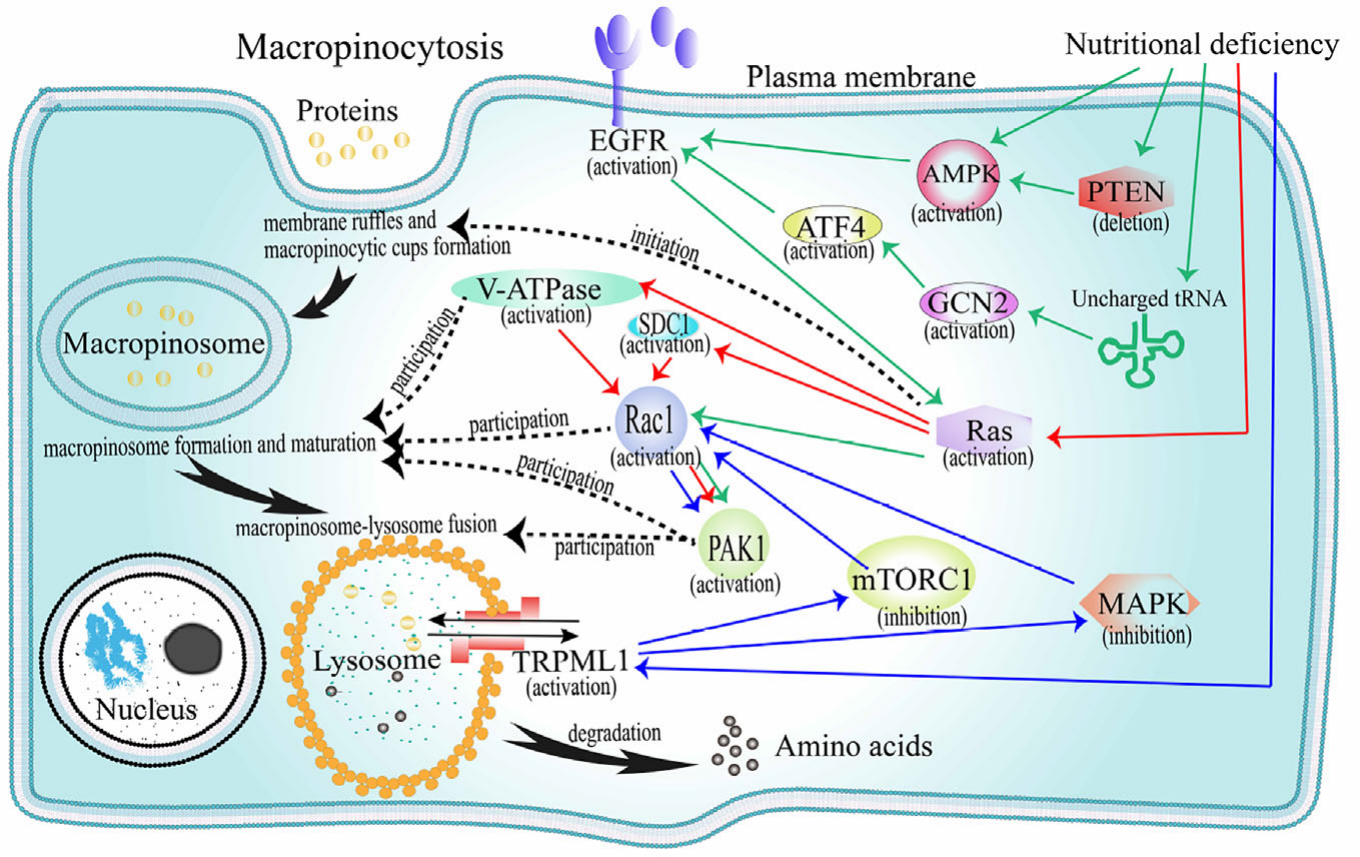
The mechanism of extracellular proteins internalization through macropinocytosis. Black solid arrow path: represents the internalization pathway of exogenous protein. The initiation protein Ras is activated, and membrane ruffles and macroinosomes begin to form. Under the regulation of V-ATPase, Rac1, PAK1, and other factors, the macropinosome matures and fuses with the lysosome, and then the foreign protein is degraded into amino acids and released. Black dotted arrow: represents the corresponding heuristic or regulatory factor corresponding to the event involved in macropinocytosis. Red solid arrow path: Under the pressure of amino acid deficiency, the Ras-V-ATPase/SDC1-Rac1-PAK1 pathway is activated and macropinocyrosis occurs. Green solid arrow path: under the pressure of amino acid deficiency, the AMPK/(PTEN-AMPK)/(GCN2-ATF4)-EGFR-Ras-Rac1-PAK1 pathway starts and macropinocytosis occurs. Blue solid arrow path: Under the pressure of amino acid deficiency, TRPML1 activates, reversely inhibits mTORC1 and MAPK, promotes the activation of Ras-Rac1-PAK1 pathway, and macropinocytosis occurs. AMPK, AMP-activated protein kinase; EGFR, epidermal growth factor receptor; V-ATPase, vacuolar ATPase; SDC1, syndecan 1; PAK1, p21-activated kinase 1; mTORC1, mechanistic target of rapamycin compound 1; MAPK, mitogen-activated protein kinase.

When cancer cells are nutrient deficient, they can obtain extracellular proteins for survival through macropinocytosis. The production of macropinocytosis that internalizes extracellular proteins mainly depends on the activation of oncogenic RAS genes and growth factor receptors (GFRs), and is negatively regulated by the mammalian target of rapamycin complex 1 (mTORC1) (**Figure 3**).

#### Macropinocytosis After Oncogenic RAS Genes Activation

Oncogenes in the RAS family (K-, H-m, and N-RAS) are the most mutated and play a key regulatory role in important signaling pathways that promote tumor growth (2, 81) (**Figure 3**). Under the pressure of nutritional deficiency, cells expressing oncogenic K-RAS can directly activate Ras protein to internalize and degrade extracellular proteins through a macropinocytic uptake mechanism (82). In recent years, increasing number of cancer cell types that express oncogenic RAS genes have been confirmed to obtain extracellular proteins through macropinocytosis in a hungry state. In pancreatic cancer cells, macropinocytosis has been shown to involve in active Ras protein signaling (17, 83). Pancreatic ductal adenocarcinoma (PDA) is observably hypervascular, and macropinocytosis is deemed to facilitate amino acid supply and survival of the tumor cells. In prostatic cancer cells, Mishra et al. found that epigenetic activation of RAS genes induced macropinocytosis involved in albumin uptake and amino acid release (primarily glutamine) to promote metabolism and differentiation of cancer cells (84). In non-small cell lung cancers (NSCLC), Hodakoski et al. confirmed a subpopulation of cell lines that can internalize and degrade extracellular proteins by macropinocytosis to maintain their survival in glucose deficient environment (85). Extracellular proteins are degraded into amino acids (86) that feed these cancer cells to meet the high nutritional requirements of cell proliferation (82, 87). For example, internalized proteins can be degraded to produce alanine, which participates in the tricarboxylic acid (TCA) cycle and the production of glycolytic intermediates. In this process, alanine transaminase 2 (ALT2) converts alanine to pyruvate, which is essential for the survival of cancer cells during glucose deficiency. Macropinocytosis in these cells is adjusted by Rac-Pak signaling activated by PI3K. Cell proliferation was inhibited when macropinocytosis was inhibited.

In recent years, research on the molecular mechanism of macropinocytosis induced by the oncogenic RAS genes has gradually intensified. Ramirez et al. proved that V-ATPase was involved in regulating RAS genes induced macropinocytosis (22). Tumorigenic RAS genes drive the V-ATPase transfer between the endomembrane and plasma membranes, which needs excitation of protein kinase A from solvable adenylate cyclase relying on bicarbonate. The collection of V-ATPase in the cell membranes facilitates the binding of Rac1 relying on cholesterol to the cell membrane, which is the primary condition for stimulating membrane ruffles and macropinocytosis. A recent study showed that macropinocytosis in PDA cells lack of glutamine needed to be driven by K-RAS gene, the activation of K-RAS gene could increase position of the transmembrane proteoglycan receptor protein, SDC1 on the plasma membrane. SDC1 could further assembly form a signaling complex that could activate Rac1 GTPase, which could stimulate PAK1 and thereby activate macropinocytosis (23). It delivered the proteins to lysosomes and degraded them into amino acids that were then used by cells.

#### Macropinocytosis After GFRs Activation

In the microenvironment of solid tumors, vascular system, fibrotic tissue, and immune cell infiltration are specific. When nutrients and growth regulators in the tumor microenvironment change, tumor cells can make adaptive responses to maintain survival. Colin et al. studied whether the expression of the key factors associated with macropinocytosis was modified in human glioma datasets (88). Thirty-eight genes associated with macropinocytosis were identified from the mRNA levels of GBM. As markers of GBM, EGFR and platelet-derived growth factor receptor (PDGFR) may trigger macropinocytosis (**Figure 3**). The stimulation of EGFR and PDGFR induces the activation of K-RAS and H-RAS gene. Lee et al. found that EGFR could regulate the interaction between macropinocytosis and nutrients in PDA (25). In the absence of glutamine, activation of EGFR signaling pathway can activate RAS genes following Rac1 and serine-threonine kinase PAK1 activation, thereby causing changes in the membrane skeleton, promoting macropinocytosis, and increasing the acquisition of glutamine. Glutamine can produce the TCA circulating intermediate, alpha-ketoglutarate, which is then demineralized to form glutamic acid. Supplementation with alpha-ketoglutarate or glutamic acid can eliminate macropinocytosis induced by glutamine deficiency. It can therefore be inferred that the decrease in glutamine metabolism, rather than the decrease in glutamine levels, is responsible for macropinocytosis. In addition, in pancreatic ductal adenocarcinoma (PDAC) cells, the lack of glutamine can lead to autophagy. This autophagy process is a macropinocytosis-associated autophagy pathway. Its activation can provide glutamine for the TCA cycle, which is beneficial to the maintenance of intracellular glutamine levels in PDAC cells (89).

There are several theories regarding the sensor that links glutamine level to the EGF signal. The first is the GCN2-eIF2α-ATF4 mechanism (90). At first, glutamine starvation results in the appearance of uncharged tRNAs that activate GCN2. Next, the level of ATF4 transcription factor increased, promoting the expression of genes related to metabolic amino acids and transduction of cytokine signals. The second is AMP-activated protein kinase (AMPK) (90). Intracellular energy levels change when TCA circulatory function is impaired and nutrient uptake is increased but utilization is reduced. In prostate cancer cells, reduced energy levels activate AMPK through direct and indirect mechanisms that induce macropinocytosis and the internalization of necrotic fragments for lipids and proteins synthesis. In addition, Kim et al. found that loss of PTEN, the most frequently deleted tumor suppressor gene in prostate cancer, can activate AMPK, which supports RAC1 and PAK1 activation and macropinosomes formation of extracellular proteins in nutritional pressure (21).

#### Macropinocytosis Negatively Regulated by mTORC1

As a nutrient-sensitive signaling protein, mTORC1 can react to environmental changes to regulate cell survival (91) (**Figure 3**). Under conditions of sufficient amino acids, the activation of mTORC1 can promote cancer cell growth. However, in a recent study, the authors found that when cells rely on exogenous proteins(through macropinocytosis) instead of exogenous amino acids as the source of amino acids, the activation of mTORC1 inhibits cell proliferation (19). Therefore, in the case of insufficient amino acids, inhibiting the activity of mTORC1 will increase the macropinocytosis of extracellular proteins to meet the needs of cell survival. Kasitinon et al. found that TRPML1, a lysosomal cation channel, whose activation under nutritional deficiency is beneficial to the occurrence of macropinocytosis and cell proliferation and survival of melanoma cells (92). The main mechanism is that TRPML1 reversely adjusts and weakens mitogen-activated protein kinase (MAPK) pathways and mTORC1 signal of melanoma cells, maintains the occurrence of macropinocytosis, and reduces or blocks protein toxic stress.

In short, oncogenic RAS genes, GFRs, and mTORC1 are of great significance for tumor cells in a nutrient-deficient environment to rely on macropinocytosis for survival. Based on this, further design of related anticancer drugs will have certain research value.

### Other Nutrients Internalization Through Macropinocytosis

**Figure 4:**
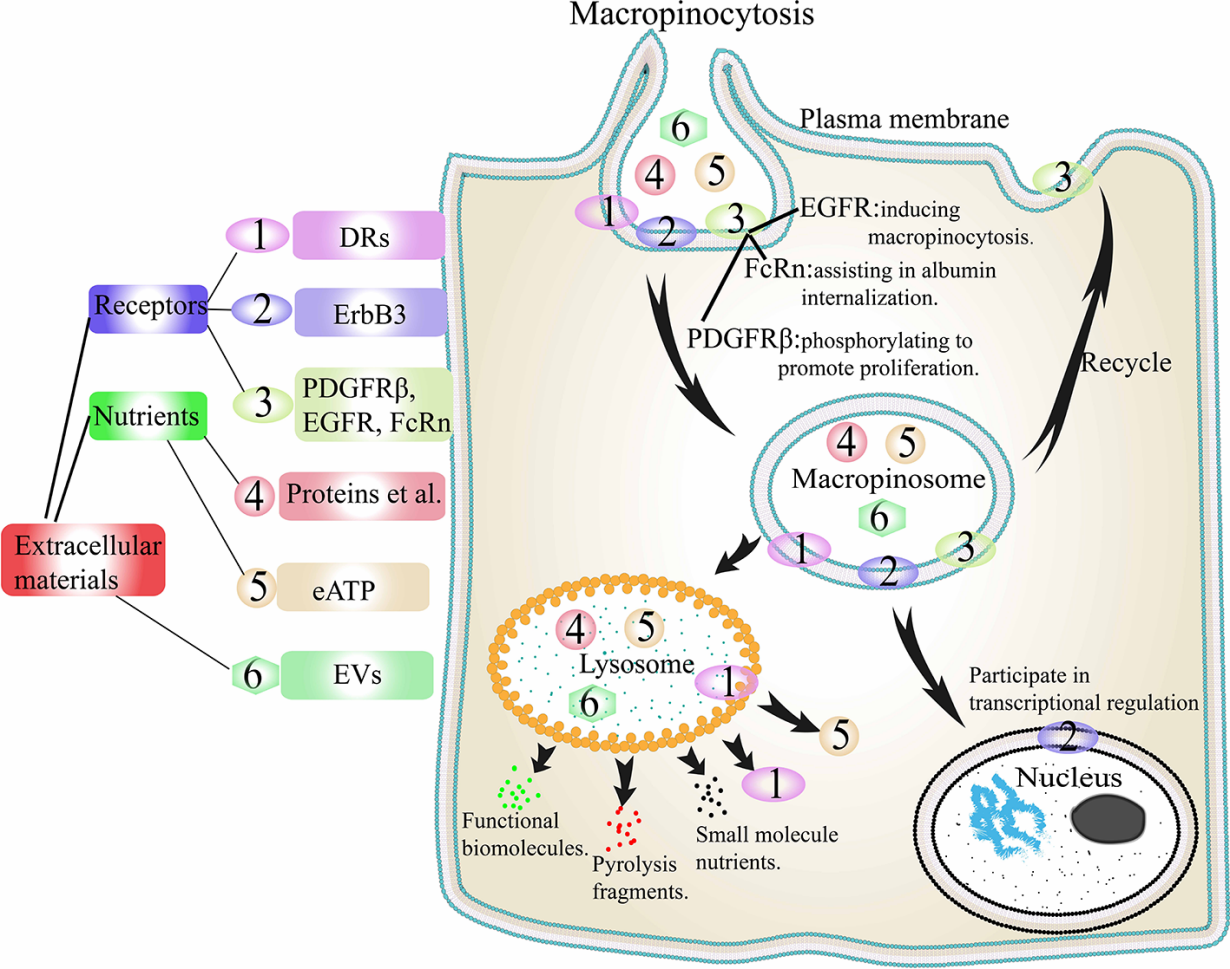
Extracellular materials internalization through macropinocytosis. PDGFR, EGFR, and FcRn are internalized by macropinocytosis to play their corresponding functions, and then cycled back to the cell surface to participate in the next macropinocytosis. DRs, EVs, proteins, eATP, and other nutrients are internalized into macropinosomes and then the macropinosome fused with lysosomes. DRs and eATP are released, EVs are degraded to release functional biomolecules, while proteins are degraded to small molecules. ErbB3 is internalized and then enters the nucleus interact with the transcription complex in the nucleus to play a role in transcription regulation. DRs, death receptors; ErbB3, a transmembrane tyrosine kinase receptor; EGFR, epidermal growth factor receptor; PDGFR, platelet-derived growth factor receptor; FcRn, neonatal Fc receptor; eATP, extracellular ATP. EVs, extracellular vesicles.

The contribution of macropinocytosis to the internalization of extracellular proteins in tumor cells has been described in detail above. Based on these, Jayashankar et al. carried out further research (93). A click chemistry research revealed that the consumption of necrotic cell debris through macropinocytosis (necrocytosis) supplied amino acids, carbohydrates, lipids, as well as nucleotides to cancer cells. Similarly, cancer cells can also drive macropinocytosis to internalize eATP to promote their own metabolic activity (18) (**Figure 4**). In the early stage of tumor cell metastasis, following internalization of eATP into cells through macropinocytosis, it induces epithelial-mesenchymal transformation (EMT) in cancer cells through purine receptor signaling, which enhances the proliferative ability of cells and their resistance to anticancer drugs (94). Cao et al. demonstrated that eATP could induce the expression of matrix metalloproteinases (MMPs) and enhance the EMT and invasions of lung cancer cells (94). This ability of eATP is partly dependent on P2X7 activation. After the internalization of eATP through macropinocytosis, early cell invasion steps related to P2X7 can be further enhanced.

### Desired Receptors Internalization Through Macropinocytosis

There are many receptors on the membrane surface in tumor cells, and internalization of some surface receptors through macropinocytosis is one way for tumor cells to maintain survival. (**Figure 4**). One is the death receptors (DRs). It has been reported that cancer cells can internalize DRs through Ras-dependent macropinocytosis, reducing the number of DRs on the cell membrane, thereby evading part of the TNF-related apoptosis-inducing ligand, TRAIL, and ultimately evading apoptosis (95). It has also been reported that even when the RAS genes are mutated, tumor cells can still internalize DRs into cells through macropinocytosis (96, 97). One is a transmembrane tyrosine kinase receptor, ErbB3. Koumakpayi and Reif et al. found that in prostate and breast cancer cells, macropinocytosis could transfer ErbB3 from the cell membrane to the nucleus to interact with the transcription complex in the nucleus to play a role in transcription regulation (98, 99). One is platelet-derived growth factor β-receptor (PDGFRβ). Schmees et al. found that in H-RAS-positive fibroblasts, macropinocytosis could internalize PDGFRβ from the cellular membrane (100). Then through macropinosomes transfer, the activity of PDGFRβ could be increased to achieve enhanced non-target-dependent cell proliferation. Another is EGFR (37). Growth factor stimulation can stimulate macropinocytosis, this type of macropinocytosis is independent of nutrients internalization. For example, in type 2 neurofibromatosis, EGF-stimulated macropinocytosis plays a major role in the rapid internalization and efficient circulation of EGFR. In addition, the neonatal Fc receptor (FcRn) can be internalized by macropinocytosis. Toh et al. demonstrated the rapid cycling pathway of FcRn ligands through macropinocytosis (101). The cells internalize albumin through macropinocytosis. When FcRn is absent in cells, albumin degrades rapidly. On the contrary, when there is sufficient FcRn in the cells, albumin will locate in the membrane region with positive SNX5 and return to the plasma membrane through early macropinosomes and microtubules. Similarly, the circulating pathway of soluble monomer IgG is similar to that of FcRn. In contrast, IgG that bind to surface Fcγ receptors and IgG that go through the macropinocytic pathway ends up in different fates.

### Beneficial EVs Internalization Through Macropinocytosis

EVs are heterogeneous phospholipid vesicles derived from various mammalian cells such as cancer cells and host cells (102). They play an important role in the process of information exchange between cells in multicellular organisms, and are the medium for cell information exchange. EVs can be roughly divided into two categories according to their biological processes: exosomes and microvesicles (103). Exosomes, with a diameter of 30–200 nm (104), are derived from the endosomal system. They are intraluminal vesicles formed by budding from the endosomal membrane of multivesicular endosomes in the process of maturation. When multivesicular endosomes fuse with the cell membrane, exosomes are secreted (105). Microvesicles, 50–1,000 nm in diameter, are vesicles formed by fission after the plasma membrane buds out, and are finally secreted into the intercellular space (106). In recent years, microvesicles have been reported to have a certain contribution to the communication of various types of cells, including cancer cells where they are usually called oncosomes (107). After separated from the donor cells, EVs carry functional biomolecules such as proteins, lipids and nucleic acids to the recipient cells to affect cell function and promote disease progression, especially malignant diseases (20, 108). Recipient cells can be reprogrammed as contributors to promote thrombosis, cancer invasion and immunosuppression to promote tumor resistance and interfere with cancer treatment (109).

In the process of EVs uptake of tumor cells, macropinocytosis pathway has been shown to have a certain contribution (110). For example, Nakase et al. found that compared with BxPC-3 cells expressing wild-type k-RAS gene, MIA PaCa-2 cells expressing oncogenic k-RAS gene exhibit strong macropinocytosis and can actively transport exosomes into cells (111). Zhao et al. confirmed that exosomes secreted by patient-derived tumor-associated fibroblasts can be taken up and supply amino acids to cancer cells lacking nutrients in a manner similar to macropinocytosis (112). At the same time, they found that wild-type k-RAS-expressed BxPC-3 cells and oncogenic k-RAS-expressed MIA PaCa-2 cells both exhibited macropinocytosis, suggesting that it does not rely on oncogenic k-RAS signaling. We can speculate that the K-RAS gene may play a role in the internalization of exosomes through macropinocytosis and the detailed mechanism and corresponding changes need to be further studied.

In addition to macropinocytosis (111, 112), the ways in which the recipient cells take up EVs(including exosomes and microvesicles) also include clathrin-dependent endocytosis (113, 114), phagocytosis (115–117), caveolae-mediated endocytosis (118) and lipid raft-mediated endocytosis (119, 120), etc. And the uptake way of EVs mainly depends on the type of donor and recipient cells. For example, PC12 cell-derived EVs can enter bone marrow-derived mesenchymal cells through clathrin-dependent endocytosis, reducing the expression level of transforming growth factor β receptor II (TGFβRII) (113). EVs from erythroleukemia cells can be internalized by phagocytic cells through phagocytosis to achieve information exchange (116). Cancer-associated fibroblasts-derived exosomes can enter pancreatic cancer cells through caveolae-mediated endocytosis to change cell metabolism and promote tumorigenesis (112). EVs from glioblastoma cells could be internalized by recipient cells through lipid raft-mediated endocytosis to transfer genetic material and signal proteins derived from tumor cells, leading to increased tumor angiogenesis and metastasis (120). Finding out the similarities and differences and detailed mechanisms of the endocytosis of different donor and recipient cells transporting EVs will be of great significance for the control and treatment of cancer in the future.

Conclusively, macropinocytosis is of great significance to tumor cells, regardless of whether they are in a state of starvation. Macropinocytosis promotes the occurrence, survival, migration, and colonization of cancer cells. Based on this, a series of treatment strategies can be designed by targeting macropinocytosis in cancer cells.

## The Harmful Effects of Macropinocytosis in Cancers—Inducing Methuosis

**Figure 5:**
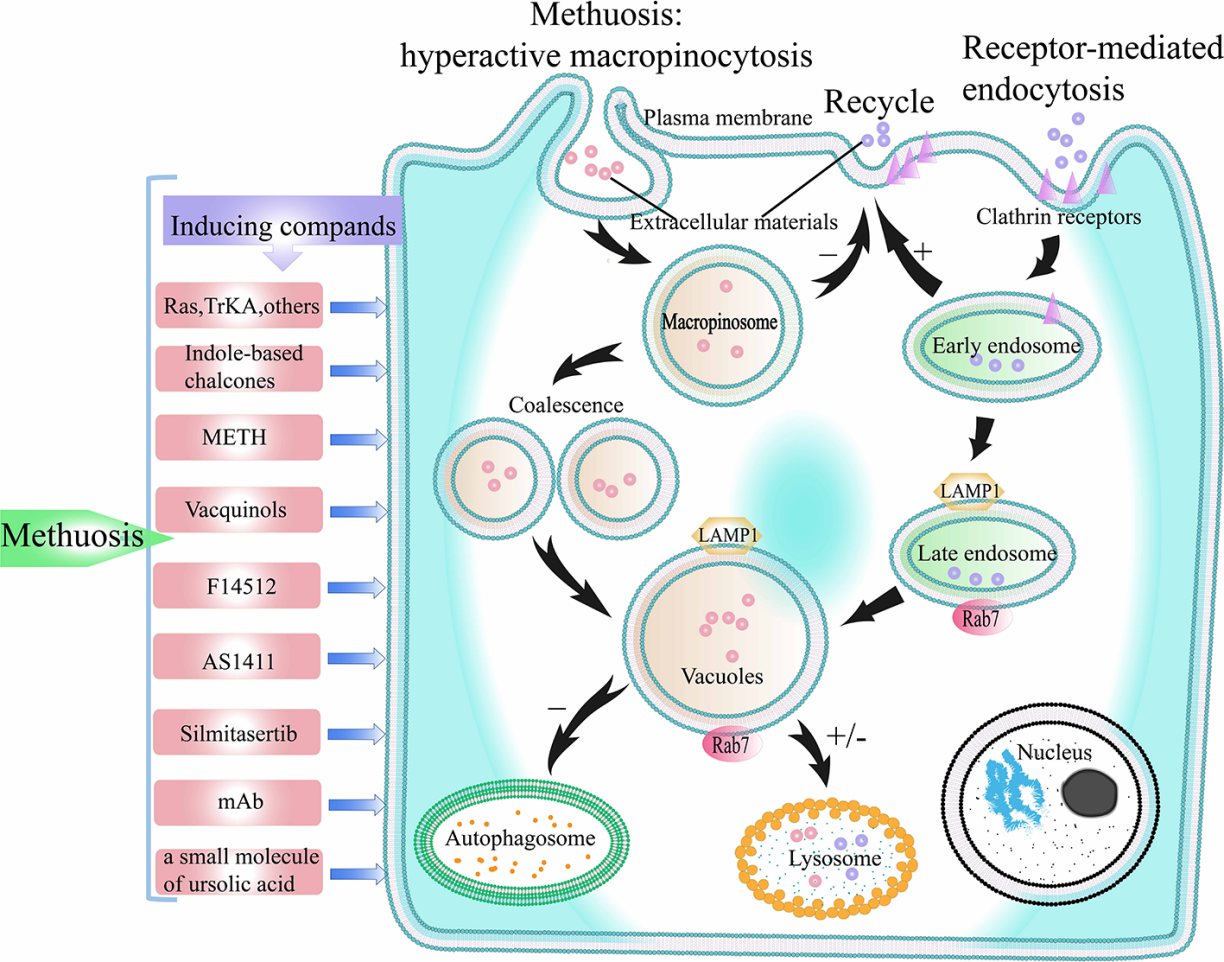
The process of methuosis. Methuosis is caused by hyperactive macropinocytosis. Macropinocytosis is a clathrin-independent endocytosis pathway. When macropinocytosis is activated, macropinosomes are produced, and macropinosomes further merge into larger vacuoles. Macropinosome does not participate in the recycling pathway of receptor-mediated endocytosis, and only few of the macropinosome is fused with lysosomes. Therefore, numerous vacuoles build up inside the cell, which eventually leads to cell death. +/- represents participating or not participating in a later pathway. TrKA, Tyrosine kinase receptor A; METH, methamphetamine.

Macropinocytosis not only promotes cancer survival but also has harmful effects on cancers. During excessive stimulation of macropinocytosis in tumor cells, the balance of macropinocytosis is disrupted; macropinosomes gradually merge with each other and the extreme vacuole formation finally leads to cell death. This novel mode of death is different from classical apoptosis and other non-apoptosis death modes such as necrosis and autophagy, and is called as methuosis (**Figure 5**). This section introduces methuosis and associated inducing factors in detail.

### Methuosis Induced by Abnormal Expression of Genes and Protein Factors

#### RAS Genes

Oncogenic mutations in RAS genes are found in about 30% of human cancers, so RAS genes are considered to be of great significance for the development of cancers. Chi et al. reported that in GBM and gastric cancer cells, abnormal activation of H-RAS oncoprotein (G12/V) was found to induce a large number of vacuoles and caspase-independent cell death (5, 15, 16). The appearance of cell vacuoles is similar to macropinocytosis. This type of cell death is called methuosis (35). In follow-up studies, methuosis has been found in other tumor cells such as osteosarcoma cells and HEK293 cells (121). This new type of cell death, which is different from apoptosis, can achieve anti-tumor effect by inhibiting the K-RAS gene mutation in normal human epithelial cells. Therefore, the over-activation of K-RAS gene has considerable promise for the treatment of cancers that can express K-RAS gene (122).

In the normal process of macropinocytosis, the plasma membrane ruffles produce macropinocytic cups, which close to form macropinosomes. After the macropinosome enters the cell, some macropinosomes circulate back to the plasma membrane surface under the action of macropinosome cyclic receptors (123, 124), while other macropinosomes fuse with lysosomes (74, 125), and are lysed (126), thereby releasing extracellular materials and nutrients (4). In GBM cells with over-activated RAS genes, the process of macropinocytosis is partly different from the normal macropinocytosis pathway. Overmeyer et al. labeled newly formed macropinosomes with fluorescent dextran and found that they did not loop back to the plasma membrane or merge with lysosomes (16). As more and more macropinosomes appeared, they began to fuse with each other to form larger macropinosomes. Following fusion, the macropinosomes exhibit the characteristics of late endosomes, which are positive for LAMP1 and Rab7. However, due to the differences between the properties of macropinosomes and advanced endosomes, some researchers believe that macropinosomes can be seen as nonfunctional advanced endosomes (16, 127). Excessive accumulation of macropinosomes results in extreme vacuolization of cells, followed by cell death.

After the onset of macropinocytosis, Ras GTPases are activated and act as mediators to promote macropinocytosis-related actin assembly (3). The key players in this process are Arf and Rab GTPases (94, 126). Over time, continuous high expressing of RAS (G12V) with subsequent slow stimulating of Rac1 causes extreme vacuolation of tumor cells, eventually leading to methuosis (128). One step that takes effect in methuosis is that the activity of macropinosomes recycled to the plasma membrane surface is reduced during the over-activated macropinocytosis process. This causes more and more macropinosomes to aggregate inside the cell, and they fuse into larger macropinosomes, eventually filling the entire space of the cell, forming extreme vacuolation and endangering cell viability. In over-activated macropinocytosis, chronic Rac1 excitement affects the other one GTPase, Arf6, which mainly causes of defects in macropinosome recycling. Arf6 plays an important role during the circulation mechanism of cytidine-uncovered endosomes (6). The authors found the active condition of endogenous Rac1 and Arf6 to be opposite. As Rac1 activation level increased, Arf6 activation was inhibited. Another important step in the process of methuosis is that the huge vacuoles formed by the fusion of macropinosomes do not fuse with lysosomes and are not degraded. Therefore, due to the lack of effective lysosomal clearance and the inability of macropinosomes to recycle to the plasma membrane, macropinosomes continue to accumulate, leading to extreme vacuolation in the cells and cell death (**Figure 5**).

#### TrkA

Tyrosine kinase receptor A (TrkA) is a receptor for neurotrophin (125), which mainly exists in endosomes induced by clathrin-dependent endocytosis and eventually degrades in the lysosome to promote the survival and differentiation of normal neuronal cells (129). In contrast, the effects of its induction on tumor cells macropinocytosis is reflected in the promotion of cell death (130) (**Figure 5**). Li et al. discovered a new mode of cell death associated with ectopically expressed TrkA activation-induced macropinocytosis in myeloma tumor cells (131). When TrkA expression was inhibited by casein kinase 1 (CK1) inhibitor, macropinocytosis and cell death were inhibited. The above-mentioned cell death is similar to methuosis induced by RAS genes overactivation. It is characterized by excessive vacuolation of macropinosomes, leading to extreme vacuolization of cells and forcing the cell to die. The most different aspect is that the macropinosomes induced by RAS genes overactivation cannot fuse with lysosomes after maturation, whereas macropinosomes induced by ectopic expression of TrkA can fuse with some lysosomes partly (35). To prove the relationship between the above mode of cell death and autophagy, Li et al. downregulated beclin-1, Atg5, LC3, and other autophagy proteins in medulloblastoma cells and found that it did not inhibit the extreme vacuolation and cell death (131). In contrast, some macropinosomes bind to LC3 protein markers and then fuse with lysosomes. It has been reported that this paradox may be due to the ability of macropinosomes to collect aberrantly expressed LC3 protein markers to regulate the downstream mechanism of the fusion process for endosomes and lysosomes (132).

#### Others

Abnormal expression and activation of genes and proteins involved in the process of normal macropinocytosis may contribute to the occurrence of methuosis. 6-phosphofructo-2-kinase/fructose-2,6-biphosphatase 3 (PFKFB3) is a key point in the glycolysis pathway. In the sarcomatoid and epithelioid cells, gene suppression of PFKFB3 can lead to methuosis (133). The inhibition of PIKFYVE (a class III phosphoinositide (PI) kinase) can also induce methuosis (134). In addition, the up-regulation of Rac1 mRNA and Rac1 protein expression can cause methuosis in human nasopharyngeal carcinoma cells (135). More detailed mechanisms and more gene and protein targets related to methuosis need further research.

### Methuosis Induced by Several Compounds

With the deepening of macropinocytosis research, it was discovered that in addition to abnormal genetic manipulations that can induce methuosis in tumor cells, trace amounts of certain drugs can also cause methuosis in tumor cells (16, 127, 133). Examples of classic drugs and emerging drugs that can induce cell death like methuosis are as follows (**Table 2**).

**Table 2:** Summary and comparison of inducing compounds of methuosis.

Inducing factors	The nature of the factors	Time for inducing macropinocytosis	Involved cancers	Relevant mechanism
Ras	Ras oncoprotein.	1-2 days	GBM and gastric cancer cells.	Continuous high expression of Ras protein and subsequent chronic stimulation of Rac1 caused extreme vacuolation of tumor cells. Macroropinosomes induced by Ras over-activation cannot fuse with lysosomes after maturation.
TrKA	Tyrosine Kinase Receptor, a neurotrophin receptor.	1–2 days	Myeloma tumor cells.	Macropinosomes induced by ectopic expression of TrkA can fuse with some lysosomes.
MIPP and MOMIPP	Indole-based chalcones.	0–4 h	U251 GBM and other cells.	Structural specificity involving structural components of the early and/or late endocytosis pathway in macropinocytosis and disturb the normal macropinocytosis process by disrupting the regulatory cycle of Rab5 GTPase.
METH	Acute and chronic abuses of psychostimulant drugs.	24 h	Neuroblastoma cells.	Involving in the activation of Ras and Rac1. The fusion of macropinosomes and lysosomes is partially impaired.
F14512	A polyamine-modified topoisomeRASe II inhibitor.	3 h	A549 NSCLC cells.	Preventing the release of intermediate DNA breaks during the action of enzymes.
AS1411	A guanine-rich oligodeoxynucleotide.	0–4 h	DU145 prostate cancer cells and Hs27 nonmalignant skin fibroblasts.	Binding to nucleoproteins and change the subcellular localization of nucleoproteins.
Silmitasertib	CX-4945, a protein kinase CK2 inhibitor.	1–2 days	Colorectal cancer cells.	Inhibiting the binding of CK2 to its Akt target, thereby inhibiting the PI3K/Akt/mTORC1 signaling pathway.
Vacquinols	A quinolone derivative.	2 h	GBM cells.	As low as 1 μM of exogenous ATP concentration could regulate Vacquinol-1-induced cell death.
mAbs	Anti-CD99 mAb.	within 15 min	Ewing sarcoma cells.	LAMP-1-positive vacuoles with CD99 and Ras/Rac1 accumulate and cause methuosis.
The pyrazole-fused ursolic acid derivatives	An ursolic acid derived small molecule.	12 h	Five human cancer cell lines.	Induces cancer cell death through hyperstimulation of macropinocytosis.

#### Indole-Based Chalcones

In 2011, researchers discovered a class of small-sized indoles, the typical representatives of which are the synthetic indole-based chalcones (MIPP, MOMIPP) (136). They can rapidly cause vacuolization of cells similar to methuosis induced by overactivation of RAS genes at very low concentrations in U251 GBM cells and a wide range of other cells (137). Using time-lapse microscopy and extracellular fluorescent yellow, the authors found that MIPP and MOMIPP rapidly induced plasma membrane shrinkage, leading to the formation of macropinosomes. The newly formed macropinosomes fuse rapidly and neither participate in membrane circulation nor undergo lysosomal fusion. MOMIPP induces inchoate disruption of amino acid assimilation and glycolytic metabolism in GBM and other cell lines (26). At the same time of these metabolic changes, the JNK1/2 stress kinase pathway is activated by MOMIPP, thus causing phosphorylation of some sites, such as Bcl-2, C-Jun and Bcl-XL. PIKFYVE (134), a class III phosphoinositide kinase, is responsible for methuosis-inducing activity. The phosphorylation of surviving members of the Bcl-2 family is mediated by JNK, and this process and interference with glucose uptake play a key role in methuosis. It has been reported that MIPP and MOMIPP have a high degree of structural specificity in inducing macropinocytosis (138), and their specific structures may be involved in the textural constitution of early or late endocytosis pathway in macropinocytosis (139). Some researchers have proposed that MIPP drugs disturb the normal macropinocytosis process by disrupting the regulatory cycle of Rab5 GTPase, thereby causing extreme vacuolation in cells and leading to methuosis (140). After treating cells with MIPP, they quantitatively measured the amount of active Rab5-GTP and Rab7-GTP on the macropinosome membrane, and found that the former was significantly downregulated, while the latter increased significantly. Based on these results, it is speculated that MIPP drugs command the components of the Rab5 complex in a direct or indirect manner.

MIPP and MOMIPP-induced methuosis have similarities and differences with Ras-related methuosis. One difference is that MIPP drugs induce macropinocytosis about 10 times faster than Ras. The macropinocytosis process induced by MIPP drugs not only disrupts the membrane recovery and fusion phase with lysosomes, but also destroys the nascent process of macropinosomes in the later stages. MIPP and MOMIPP also have a characteristic manifestation in that the macropinocytosis induced in a short time (4 h) is reversible (35). Another difference is that the target points of action of MIPP-induced macropinocytosis are the downstream endocytosis factors of Rac1 and Arf6 rather than Rac1 and Arf6 that are acted upon by Ras protein. To study the mechanism of MIPP-induced macropinocytosis, researchers treated cells with bafilomycin A1 (Baf-A) combined with MIPP, and found that Baf-A not only increased the lysosomal pH value, but also interfered with the conversion in early endosomes and late endosomes (141). The potential molecular mechanism of methuosis needs further understanding, and the application of MIPP compounds in clinical anti-tumor therapy has promising prospects (142).

#### Methamphetamine

The main role of well-known methamphetamine (METH) is to alter the integrality of dopamine teleneurons and disrupt nerve conduction. METH has been reported to cause vacuolation of lysosomes (143). In studying the neurotoxicity of METH, Nara et al. found a specific cell death manner like methuosis (144). They cultured neuroblastoma cells in METH for 24 h and found that macropinocytosis appeared, followed by cell death. The author traced the liquid in the macropinosomes induced by METH, and observed that the macropinosomes did not arise from the endoplasmic reticulum or autophagosome, and the source was like that of RAS genes-induced and MIPP-induced macropinosomes. At the same time, in the process of macropinocytosis induced by METH, there was also a disorder of fusion of macropinosomes and lysosomes. In 2012, Nara et al. demonstrated that METH-induced macropinocytosis involved the activation of Rac and Rac1 (145). To study the correlation between Ras and Rac1 activation and METH-induced macropinocytosis, the authors also treated the cells with inhibitors of Ras and Rac and found that the production of macropinosomes was inhibited. During the formation of the macropinosomes, the lysosomal function was defective, and the authors speculated that the defective lysosomal function may be the cause of cell death. Nara et al. found that in METH-induced macropinocytosis, some FITC-dextran-labeled vacuoles co-localized with LAMP1, indicating fusion of macropinosomes and lysosomes (145). Meanwhile, it has been reported that LAMP1 can also be detected on the membranes of advanced endosomes and mature macropinosomes (125). It is speculated that the cause of cell death is an impediment to the transport of mature macropinosomes to the lysosome.

The behavior of METH-induced macropinocytosis is different from that induced by RAS genes and MIPP. For instance, there is no caspase activation-induced cell death in the process of METH-induced macropinocytosis. In cells where RAS genes are over-activated and treated with MIPP, caspase activation-induced cell death coexists with methuosis; however, methuosis dominates between these two types of cell deaths (35). Autophagosomes also exist in the process of METH-induced methuosis. However, macropinosomes induced by RAS genes overactivation are not associated with autophagosomes. In their study, Nara et al. used an autophagy inhibitor to inhibit the methuosis induced by excessive activation of RAS and observed that it did not prevent cell death (144).

#### Vacquinols

Vacquinol-1 (146), a quinolone derivative, shows promising therapeutic potential by inducing rapid cell death in GBM. Vacquinol-1-induced cell death is similar to methuosis (147). Vacquinol-1 has well drug metabolism capability in both vascular tissues and brain tissues. In animal models of GBM multiform, treatment with Vacquinol-1 has been shown to prolong patient survival. In a major finding, as low as 1 μM of exogenous ATP concentration could regulate Vacquinol-1-induced cell death (148). A study found that Z−VAD−FMK, a specific caspase inhibitor, can inhibit the function of hepatocellular carcinoma cell death caused by Vacquinol-1, which suggests that cell death caused by Vacquinol-1 is related to apoptosis (89). Therefore, the specific types of vacuolation and cell death induced by Vacquinol-1 are controversial, and more evidence is needed.

#### Others

AS1411 is an anticancer drug that specifically disturbs growth and mediates death in cancer cells. Its essence is a guanine-rich oligodeoxynucleotide, which can be used as an aptamer to bind to nucleosides (149–151). Reyes-Reyes et al. found that in cancer cells treated with AS1411 (152), the drug binds to nucleoproteins and changes the subcellular localization of nucleoproteins (153), causing cell swelling and cytoplasmic vacuoles, leading to excessive macropinocytosis similar to methuosis (154).

F14512, a cancer cell-targeting drug, is essentially a polyamine-modified topoisomerase II inhibitor (155). The anticancer mechanism of F14512 involves killing of cancer cells by blocking the liberation of intermediate DNA breakage during the action of the enzyme (156). F14512 has been reported to have anticancer properties resulting from the accumulation of numerous multilamellar vesicles and vacuoles like methuosis (157). However, unlike classic methuosis, in the cytotoxic process induced by F14512, the cells enter a doddery status as revealed by β-galactosidase dye. Therefore, the relationship between F14512-induced cytoplasmic vacuolation in the cancer cells and methuosis needs further studies.

As a silmitasertib precursor, CX-4945 can inhibit the activity of a protein kinase—CK2. Lertsuwan et al. demonstrated that CX-4945 can inhibit cell survival and induce cell death like methuosis through CK2-independent passages in cholangiocarcinoma and other cancer cell lines (158). In colorectal cancer cells, Silmitasertib inhibits the binding of CK2 to its target, Akt, thereby inhibiting the PI3K/Akt/mTORC1 signaling pathway, and tumor growth (159). During Silmitasertib treatment, tumor cells produce numerous catastrophic cytoplasmic vacuoles like methuosis, which was revealed by molecular markers. But the specific mechanism needs further research.

CD99 is one of the target molecules of Ewing sarcoma (EWS) (160). CD99 triggered by mAb can cause IGF-1R/Ras/Rac1 complex and produce Rab5-positive endocytic vesicles. In subsequent pathways, the IGF-1R/Ras/Rac1 complex dissociates and the IGF-1R circulates back to the membranes. Ras/Rac1 and CD99 continue to exist on the isolated immature LAMP-1-positive vacuoles, which would continue to accumulate and cause methuosis. Therefore, CD99 targeted therapy has great potential in the treatment of EWS patients with resistance to apoptotic drugs.

Sun et al. reported a small molecule derived from ursolic acid that may mediate cell death by macropinocytosis overactivation (161). The death was prevented after amiloride treatment, a specific inhibitor of macropinocytosis, suggesting that the cell death pattern caused by this small molecule is consistent with methuosis.

In conclusion, as a new form of cell death, methuosis needs further studies. Although the importance of RAS genes and TrkA is known, the molecular mechanisms associated with methuosis remain unclear. In addition, there are more stimulants of methuosis waiting to be discovered for clinical applications.

## Anticancer Therapies Targeting Macropinocytosis in Cancers

Due to the universality of macropinocytosis in some cancers, anti-cancer therapy targeting macropinocytosis has become an area of extensive researches. This section discusses the potential of macropinocytosis as a target for anticancer therapies from the following two perspectives: the delivery of anticancer drugs and the destruction of macropinocytosis.

### Delivery of Anticancer Drugs Through Macropinocytosis

#### Drug Conjugates Delivered Through Macropinocytosis

**Figure 6:**
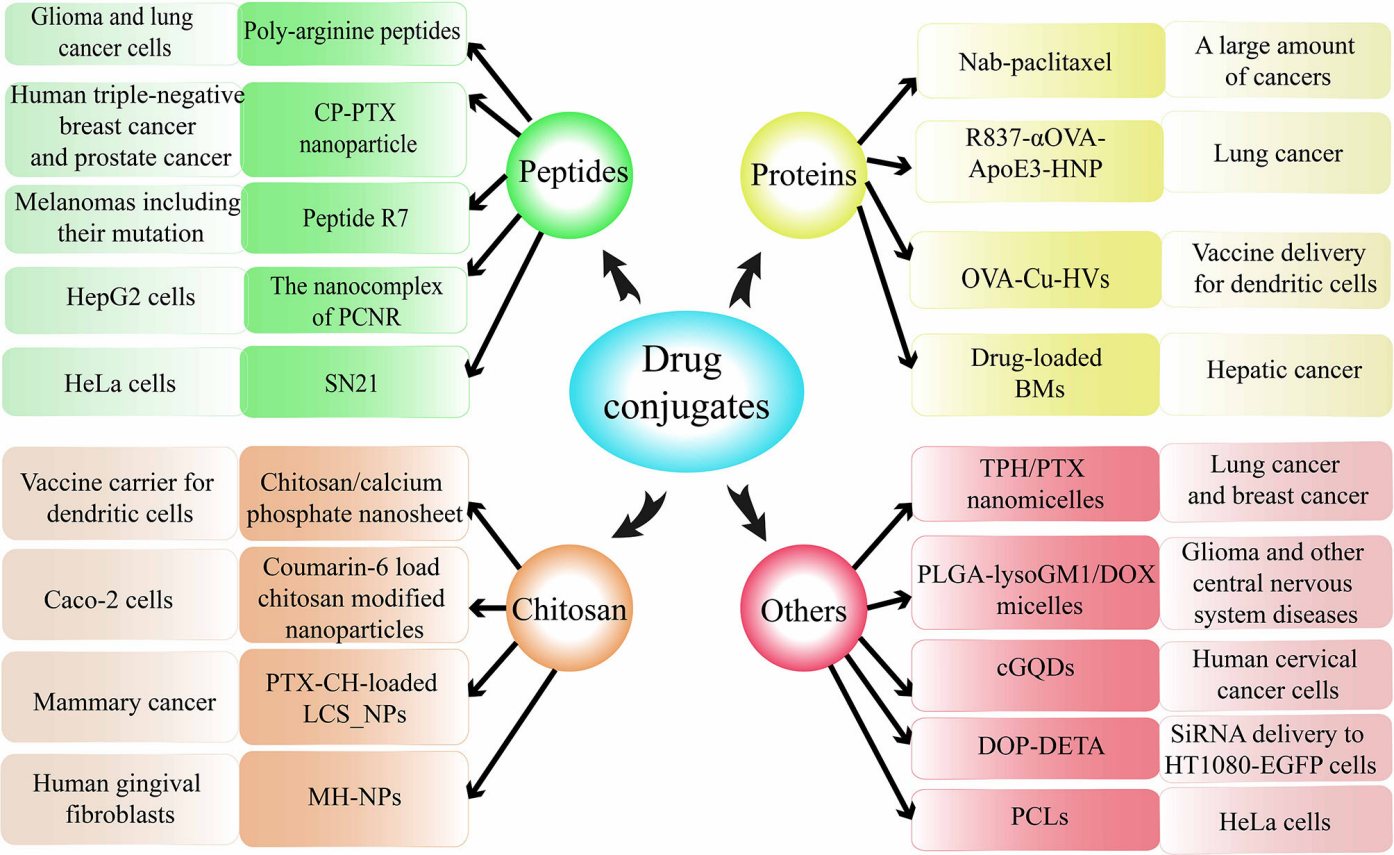
Drug conjugates delivered through macropinocytosis.

As mentioned earlier, some drugs have the ability to induce macropinocytosis, but they are only a minority. Therefore, researchers have begun to shift their focus to the combination of substances that can activate cell surface receptors of macropinocytosis and non-macropinocytosis-dependent anticancer drugs. Through the formation of this kind of drug conjugates, the macropinocytosis pathway is used as a way of anticancer drug presentation to promote cancer treatment (162). Here, the drug conjugates that can cause macropinocytosis are introduced as follows according to the types of some raw materials (**Figure 6**).

##### Peptides

Poly-arginine peptides, a classic substance for drug coupling, could achieve increased delivery of anticancer drugs through macropinocytosis in cancers (163–165). CP-PTX, formed by paclitaxel (PTX) and recombinant chimeric polypeptides (CPs) conjugation, can spontaneously self-assemble into nanoparticles (NPs) and can be internalized into tumor cells through macropinocytosis (166). PCNR, a NP based on RGD (cyclic Arg-Gly-Asp peptide) has great potential in the treatment of melanomas. And one of the PCNR uptake pathways is mostly macropinocytosis-dependent in A375 cells (167). Heptapeptide R7, a novel peptide, was synthesized to promote internalization of chlorin e6(a photosensitizer) into HepG2 cells by activation of endocytosis and/or macropinocytosis in comparison with initial NPs without peptide R7 (168). SN21, a peptide based on stromal-derived factor 1α, can induce macropinocytosis and deliver functional siRNA and proteins such as antibodies and Cre recombinases (128).

##### Proteins

Albumin was observed to accumulate in certain tumor cells through the action of macropinocytosis (169). Therefore, the design of albumin-coupled cytotoxic drugs can be used for the presentation of anti-cancer active ingredients (170). Nab-paclitaxel, a typical coupling drug in a nano-albumin-bound form causing macropinocytosis, has been applied in the cure of a large amount of cancers. R837-αOVA-ApoE3-HNP, a bionic nano vaccine, one of its components is a membrane phospholipid loaded with antigen ovalbumin (OVA), and it can use macropinocytosis to facilitate antigen presentation of DCs (171). OVA-Cu-HVs, a hybrid vaccine composed of antigen ovalbumin (OVA) and copper (II) sulfate(Cu), was manufactured to serve as a new vector related to macropinocytosis (172). DBMs, drug-loaded anthracycline-loaded bacterial magnetosomes with enhanced anticancer efficiency, can be internalized into HepG2 cells through caveolae-mediated macropinocytosis and endocytosis (173).

##### Chitosan

Chitosan calcium phosphate nanosheets, a vaccine vector consist of CaHPO_4_·2H_2_O crystals, can be swallowed by DCs through macropinocytosis to increase efficiency (174). Chitosan-modified NPs, loaded with coumarin-6, was found to undergo clathrin-mediated endocytosis and macropinocytosis, and demonstrated excellent ability to improve oral drug delivery and cellular uptake (175). PTX-CH-loaded LCS_NPs, containing paclitaxel, cholesterin, lecithin, and chitosan, can effectually enter cells by macropinocytosis and could be applied in alleviative therapy by injecting, showing reformative security as well as anticancer potency (176). MH-NPs, prepared with chitosan-NPs and minocycline, can be internalized through macropinocytosis to exert antibacterial and antiphlogistic effects (177).

##### Others

TPH/PTX nano-micelles, consist of TPP-Pluronic F127-hyaluronic acid (TPH) and paclitaxel, revealed notable cancer targeting and powerful anticancer effect by entering acidic lysosomes through macropinocytosis in breast cancers (178). PLGA-lysoGM1/DOX nano-micelles, a new nano-carrier, exhibited efficient cell absorbing through macropinocytosis, autophagy/lysosomal approach, and immense potential to treat neurologic diseases such as glioma (179). cGQDs, carboxylated graphene quantum dots, entered human cervical cancer cells mainly through the macropinocytosis-dependent pathway and showed outstanding antitumor ability and low systemic toxicity (180). DOP-DETA-based liposomes, a special lipid derivative response to pH, could transmit siRNA through macropinocytosis, and mediate RNA interference under low siRNA conditions (181). In addition, PCLs, plier-like cationic liposomes, was found primarily internalized through macropinocytosis, thus, have great prospects as alternative effective gene delivery systems (182).

Therefore, the absorption of drug conjugates by tumor cells in the form of macropinocytosis and their absorption efficiency are related to the composition (183). Besides, the size (184), structural characteristics (185), concentration (186) and clearance of the conjugates (187) also play a role. In future research, the design of drugs should take the above factors into consideration to increase the drug intake in tumor cells.

#### The Modified EVs Internalized Through Macropinocytosis

Based on the role of EVs (including exosomes) in cell information communication, the potential of EVs as drug carriers has been discovered (108). Takenaka et al. reported that EVs as carriers of gefitinib can improve the therapeutic effect of EGFR-mutant NSCLC tumor patients through macropinocytosis (104). Nakase et al. designed EVs loaded with ribosome-inactivating protein saporin with EGF that can produce stronger tumor suppression (111). Subsequent studies found that EVs modified with arginine-rich cell penetrating peptide can induce macropinocytosis and promote the delivery of saporin (188). Particularly, engineered exosomes (iExosomes), also known as mimic exosomes, are artificially designed exosomes carrying functional molecules or drugs that are used for cancer treatment. Lou et al. confirmed that iExosomes loaded with miR-199a derived from mesenchymal stem cells can enhance the sensitivity of liver cancer to chemotherapy drugs by targeting the mTOR pathway (189). Kamerkar et al. reported that artificially designed iExosomes from normal fibroblast-like mesenchymal cells carrying short interfering RNA or short hairpin RNA specific to oncogenic KrasG12D can be internalized by macropinocytosis and inhibit the development of pancreatic cancer in mice (104). As carriers, iExosomes can also be used in the treatment of neurological diseases such as schwannoma, glioma and Parkinson’s disease (190). Targeted cells can absorb EVs through a variety of endocytosis pathways, including macropinocytosis, caveolin-mediated uptake, phagocytosis, and lipid raft-mediated endocytosis, etc. (191, 192).

In conclusion, macropinocytosis for delivering antitumor drugs holds immense potential for research and clinical implications. Macropinocytosis can present substances including monomer drugs, drug NPs and other drug conjugates, and EVs. Through macropinocytosis, antitumor drugs can quickly and efficiently enter tumor cells to exert antitumor effects.

### Tumorigenesis Suppress Following Macropinocytosis Destruction

**Figure 7:**
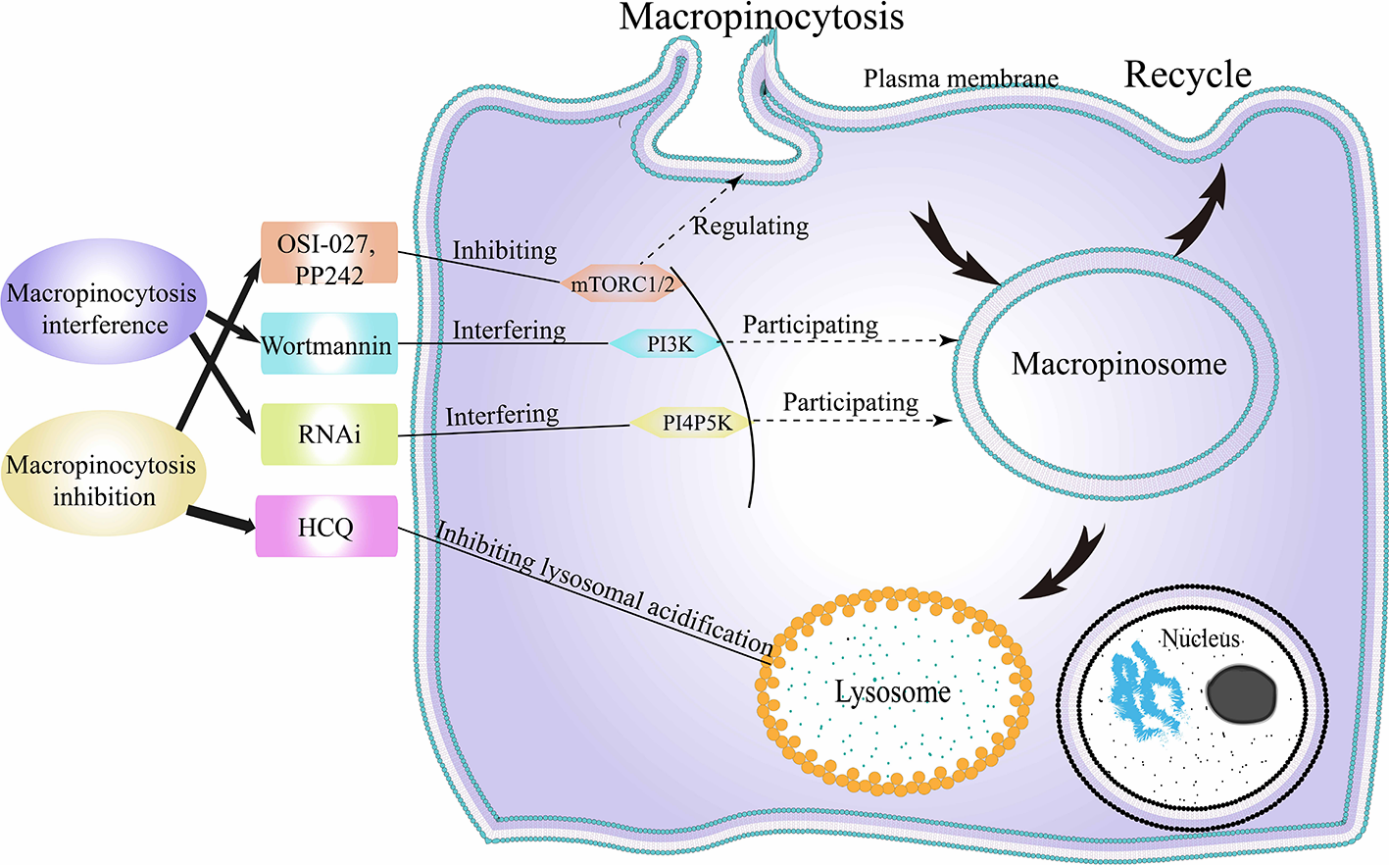
Cancer suppression following macropinocytosis destruction. HCQ, hydroxychloroquine; RNAi, RNA interference; mTORC1/2, mechanistic target of rapamycin compound 1/2; PI3K, class-I phosphatidylinositide 3-kinase; PI4P5K, phosphatidylinositol 4-phosphate 5-kinase.

As mentioned above, cancer cells can utilize macropinocytosis to internalize what they need or lack, to promote their own proliferation and metabolism. Therefore, some researchers are attempting to arrest normal macropinocytosis activity in cancer cells to achieve growth suppression. Current research in this area focuses on the following two aspects (**Figure 7**).

#### Cell Metabolism Reducing Under Macropinocytosis Inhibition

The first point is to inhibit tumor metabolism by inhibiting macropinocytosis in tumor cells (193) (**Figure 7**). When cells are in an amino acid-deficient environment, tumor cells upregulate macropinocytosis. Macropinocytosis, in turn, promotes the endocytosis of extracellular proteins by affecting mTORC1 in cancer cells, thereby maintaining tumor cell survival (194). It has been reported that hydroxychloroquine (HCQ) can indirectly inhibit macropinocytosis in tumor cells by inhibiting lysosomal acidification (195). Following inhibition of macropinocytosis, the ability of tumor cells to metabolize extracellular proteins and eATP is weakened, and the lack of metabolic substrates limits the viability of tumor cells (196). The mTOR pathway is often disrupted in cancers, and the intersection between mTOR and TPCs suggests intriguing therapeutic possibilities (197). In cancers associated with skin, breast, lung and cervix, and rhabdomyosarcoma (RMS), Srivastava et al. found that the use of dual mTORC1/mTORC2 inhibitors, OSI-027 and PP242, resulted in over-activation of macropinocytosis, inhibiting tumor survival (24). In further experiments, xenograft RMS in mice treated with OSI-027 showed reduced tumor growth, reduced proliferation, and excessive macropinocytosis in cancer cells. These results suggested that dual inhibitors may be useful in the treatment of refractory or recurrent cancers.

Identifying the adaptive metabolic pathways in cancers may provide novel targets for cancer therapy. RAS genes-induced macropinocytosis of extrinsic proteins in glucose-deficient tumor cells, an adaptive metabolic pathway for cancers, may be a potential target for anticancer strategy (85). Liu et al. demonstrated significantly greater uptake of NPs by cancer cells that have activating mutations of K-RAS gene (86). Such an NP-based therapy that targets K-RAS gene-induced macropinocytosis is a handy way toward improved transmitting into K-RAS gene-induced tumors. Research on the inhibition of macropinocytosis in this strategy may contribute to the improvement of the curative effect of cancers (198). Similarly, Ramirez et al. found that V-ATPase, as one of the regulators of RAS genes mediated macropinocytosis, is closely related to nutritional supply (22). Thus, V-ATPase inhibitors may inhibit the metabolic adaptation of RAS genes mutant cancer cells.

#### Cell Survival Inhibition Under Macropinocytosis Interference

The second point is to make macropinocytosis abnormal through the interference of the physiological process of macropinocytosis, thereby incapacitating it to help cancer cells absorb beneficial substances. (**Figure 7**). Currently, most of the research in this area is centered around interfering with macropinocytosis by interfering with the phosphoinositide biochemical pathway. Thapa et al. reported that when Iγi2, a specific PI4P5K, was knocked down using RNA interference (RNAi) in breast cancer cells, the viability of the breast cancer cells was suppressed (199). Similarly, Araki and Teranishi et al. found that in pancreatic cancer cells, the process of macropinocytosis was disrupted following treatment with the fungal metabolite, wortmannin, which blocks PI3K (200, 201). Consequently, the viability and metastasis of the pancreatic cancer cells were inhibited. Salloum et al. found that macropinocytosis induced by tyrosine kinase receptors excitement was strongly relying on a PI3K subtype, PI3Kβ (202). In the early stage of macropinocytosis, PI3Kβ is involved in regulating the formation of circular folds. In the late stage of macropinocytosis, PI3Kβ participates in the activation of Rac1-activated downstream molecules. Similarly, PI3Kβ is involved in regulating macropinocytosis in tumor cells with defective PTEN tumor suppressor genes. Therefore, pharmacological inhibitors of PI3Kβ may have great potential in anticancer treatment. In addition to the phosphoinositide pathway, methods to interfere with other pathways of macropinocytosis remain to be studied, and their significance in tumor treatment will become increasingly clear in the future.

In GBM cells, the presence of macropinocytosis is conducive to drug presentation, and persistent macropinocytosis may eventually cause methuosis. Therefore, Colin et al. hypothesized that the combined use of macropinocytosis inducers might achieve the dual effects of drug presentation and methuosis promotion (88). SN21 was combined with membrane cleavage peptides by Arafiles et al. to realize the internalization through macropinocytosis and release of endosomes of extracellular materials (128). This strategy has been shown to be effective in promoting cellular uptake of anticancer drugs. Cao et al. found that eATP uptake by macropinocytosis is involved in early metastasis steps such as EMT of tumor cells (94). Tumor survival was inhibited in nude mice with macropinocytosis-associated SNX5 gene knockout. These results demonstrate that the importance of eATP in tumor metastasis, suggesting that intracellular energy balance biochemical equation requires new consideration, and indicating that eATP internalization through macropinocytosis may be an effective anticancer drug therapy target.

In short, whether the inhibition of macropinocytosis is achieved through inhibition of the macropinocytosis process itself, or through the associated inducible and regulatory factors, as long as the macropinocytosis activity in cancer cells is inhibited, it may interfere with the survival of the cancer cells and inhibit cancer. Therefore, elucidating the molecular mechanism of macropinocytosis in cancer cells is of great significance for cancer therapy.

## Summary and Outlook

Macropinocytosis is a clathrin-independent pathway of endocytosis of extracellular materials. Macropinocytosis has great research value for tumor survival and treatment. On the plus side for cancer growth, macropinocytosis is an important approach through which cancer cells intake extracellular nutrients (such as proteins), receptors, and EVs. What happens in this case is the constitutive macropinocytosis, which will not cause cell death. Constitutive macropinocytosis is a spontaneous response of tumor cells in response to changes in the surrounding environment based on the need for survival. Taking protein intake as an example, constitutive macropinocytosis mainly occurs in tumor cells that can express the RAS genes or cells lacking the PTEN gene under undernourishment conditions. In this case, the pressure of undernutrition is the main cause of constitutive macropinocytosis (203). On the down side for cancer survival, overactivated macropinocytosis is able to induce a new type of non-programmed cell death in cancer cells, called methuosis. In tumor cells that can develop constitutive macropinocytosis, genetic interference with the expression of related genes and proteins (such as Ras, TrKA) involved in constitutive macropinocytosis, and drug treatment (such as MOMIPP) both can induce methuosis associated with hyperactivated macropinocytosis. The macropinocytosis that occurs in this case is excessively activated, which is different from the constitutive macropinocytosis. In constitutive macropinocytosis, part of the mature macropinosomes are fused with lysosome and then degraded, and the others recycle back to the plasma membrane surface and fuse with the membrane. However, in the over-activated macropinocytosis, the macropinosome-lysosome fusion and the macropinosome cycle back to the plasma membrane rarely occur, which will cause a large number of macropinosomes accumulating in the cells and eventually lead to cell collapse and death. In view of the beneficial and harmful effects of macropinocytosis on cancer cell growth, researches into the design of anticancer therapies targeting macropinocytosis have made substantial progress, including the utilization of macropinocytosis to deliver anticancer drugs, abrogation of macropinocytosis to suppress cancers, and designing anticancer drugs that induce methuosis. However, there are still challenges and questions about the research in this field.

Firstly, methuosis is proposed as a new non-apoptotic form of cell death that is different from paraptosis, autophagy, necrosis or oncosis. However, there is still controversy about this point of view. A potential point of contention is whether methuosis really represents a unique form of regulating cell death, or is it just a subtype of necrosis or oncosis (35). To prove that methuosis is indeed a new and unique way of cell death, there are still many problems to be solved. One of them is concrete evidence of the connection between vacuolation and cell death. In the occurrence of methuosis, all or most of the macroinosomes do not fuse with lysosomes, and all or most of the macroinosomes do not circulate back to the surface of the plasma membrane. These two phenomena have major contributions to the extreme vacuolation of cancer cells. Some scholars believe that the emergence of extreme vacuolation led to the death of cancer cells (128, 140). However, more detailed, and specific evidence linking macropinocytosis directly to cell death is lacking. The specific mechanism and molecular pathways between cell death and extreme vacuolation are still unclear. In addition, the pathophysiological manifestations and specific cell death procedures involved in methuosis also require clearer understanding and more in-depth researches. The unique pathophysiological manifestations and death procedures different from other non-apoptotic cell deaths may provide key evidence for the argument that methuosis is a new type of cell death.

Additionally, macropinocytosis, the dual role of promoting cell growth and participating in cell death, can occur in the same type of cells. This means that perhaps macropinocytosis has a “threshold” between promoting cell survival and death. On one side of this “threshold”, macropinocytosis fully or mainly plays a role in promoting cell survival, while on the other side, macropinocytosis is fully or mainly involved in cell death. For example, for cancer cells that can express the RAS genes, when the RAS genes are normally activated, constitutive macropinocytosis will occur to promote cell survival (17, 83). However, when the RAS genes are over-activated, it will lead to the occurrence of methuosis (122). Perhaps there is a “threshold” for the activation of the RAS genes. When the activation level of the RAS genes is on different sides of the “threshold”, it may cause beneficial or harmful effects on cancer cells. In addition, in the study of small-molecule drugs that induce methuosis, contrary to the phenomenon that high-concentration drugs promote cancer cell death, extremely low-concentration drugs can promote the proliferation of cancer cells (158),, which may also indicate the existence of a “threshold” in macropinocytosis that promotes cell survival and participates in methuosis. However, what exactly this “threshold” is needs to be further studied, and the specific mechanisms and key pathways of different functions that exist on both sides of this “threshold” of macropinocytosis also need to be discovered.

## Author Contributions

SS wrote the manuscript. YZ collected the literature and generated the figures and tables. TD edited and checked the manuscript format. NJ and HZ reviewed the manuscript. All authors contributed to the article and approved the submitted version.

## Funding

This work was supported by the National Natural Science Foundations of China (No. 81922020, 81970950), the Postdoctoral Research and Development Funding of Sichuan University (2020SCU12016), the Research Funding for Talents Developing, West China Hospital of Stomatology Sichuan University (No. RCDWJS2020-4, RCDWJS2020-14), and the CAMS Innovation Fund for Medical Sciences (CIFMS,2019-I2M-5-004).

## Conflict of Interest

The authors declare that the research was conducted in the absence of any commercial or financial relationships that could be construed as a potential conflict of interest.
